# 10d, a Pyridoquinoxaline-Based P-Glycoprotein Inhibitor, Exacerbates MPTP-Induced Neurotoxicity in PC12 Cells

**DOI:** 10.3390/cimb48090912

**Published:** 2026-09-05

**Authors:** Claudia Cannas, Gaia Rocchitta, Antonio Carta, Sandra Piras, Rossana Migheli

**Affiliations:** Department of Medicine, Surgery and Pharmacy, University of Sassari, Viale San Pietro 43/b, 07100 Sassari, Italy; c.cannas1@studenti.uniss.it (C.C.); grocchitta@uniss.it (G.R.); acarta@uniss.it (A.C.); piras@uniss.it (S.P.)

**Keywords:** pump inhibitor, P-glycoprotein, dopamine, PC12 cells

## Abstract

Efflux pumps are essential components of cellular detoxification mechanisms, regulating the intracellular accumulation of xenobiotics and endogenous compounds. Among them, P-glycoprotein (P-gp) plays a role in protecting the brain from potentially toxic molecules, and alterations in its function have been associated with neurodegenerative disorders, including Parkinson’s disease (PD). Although P-gp inhibitors have been extensively investigated in the context of multidrug resistance, their effects on neuronal cells remain poorly characterized. In the present study, we investigated the biological effects of the pyridoquinoxaline-based efflux pump inhibitor 2,2′-(pyrido[2,3-g]quinoxaline-2,3-diylbis(methylene))bis(oxy)bis(N-phenylbenzamide) (**10d**) in PC12 cells, a widely used dopaminergic neuronal model. The effects of **10d** were evaluated by an MTT-based cell viability assay, while intracellular and extracellular dopamine (DA) levels and DA metabolites were quantified by high-performance liquid chromatography (HPLC). In addition, the ability of **10d** to modulate MPTP-induced neurotoxicity was assessed, alone and in combination with amantadine (AMA), a known antiparkinsonian drug. Exposure to **10d** (5 and 10 μM) reduced PC12 cell viability and markedly enhanced MPTP-induced cytotoxicity. Furthermore, **10d** altered dopaminergic homeostasis by decreasing intracellular DA levels and modifying DA metabolite profiles, with more pronounced effects following co-treatment with MPTP. The combined administration of **10d**, MPTP and AMA produced a stronger disruption of DA metabolism compared with individual treatments or **10d**/MPTP co-exposure.

## 1. Introduction

**Figure 1:**
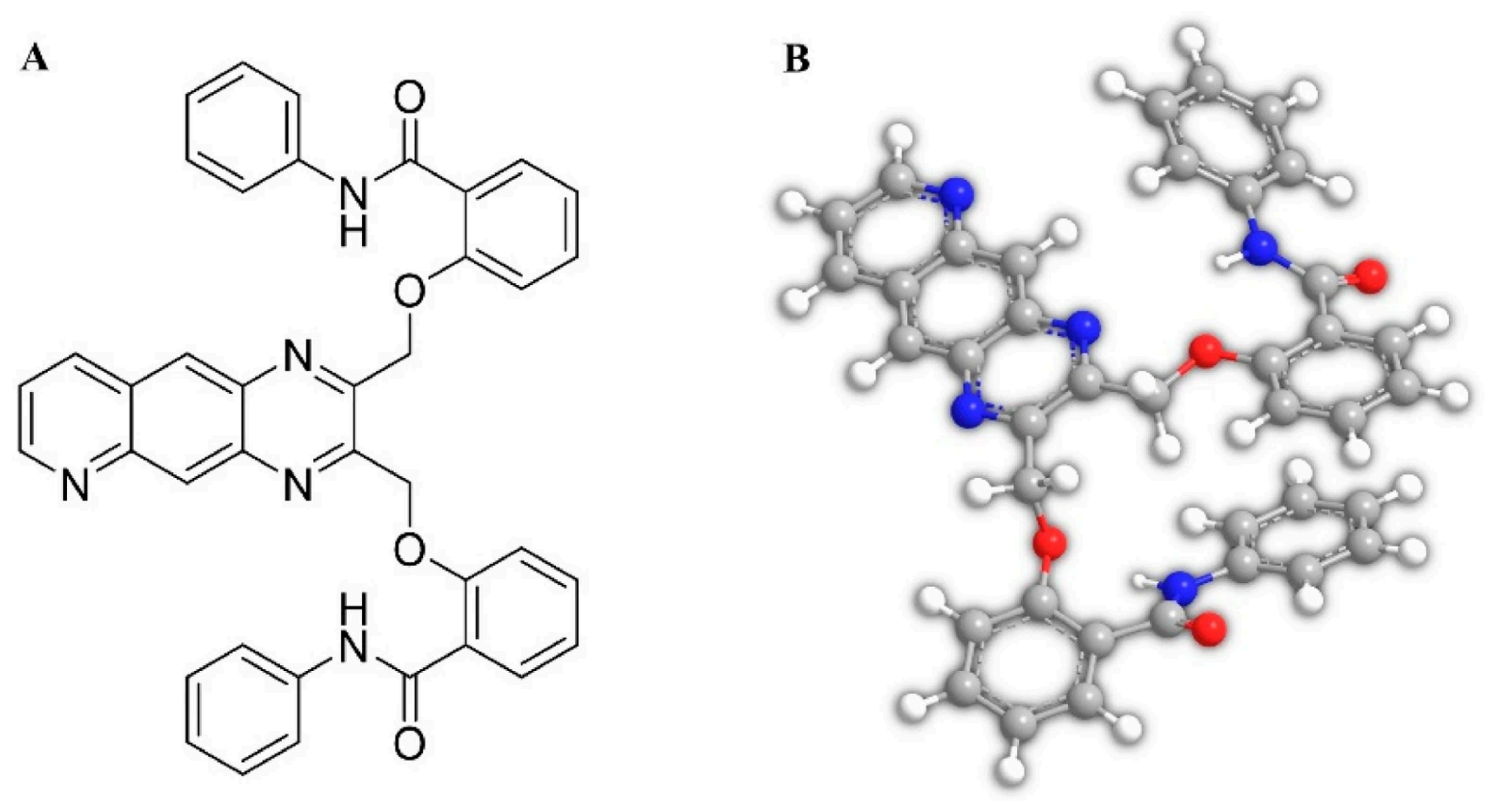
Chemical structure of 2,2′-((pyrido[2,3-g]quinoxaline-2,3-diylbis(methylene))bis(oxy))bis(N-phenylbenzamide). (**A**) Two-dimensional representation. (**B**) Three-dimensional ball-and-stick model with CPK atom coloring (C, gray; N, blue; O, red; H, white).

The role of efflux pumps in the extrusion of exogenous substances and in drug resistance has been extensively documented [1]. By actively extruding a wide range of compounds, these transporters contribute to multidrug resistance in both cancer cells and pathogenic bacteria, while also modulating the plasma and tissue concentrations of endogenous molecules and xenobiotics, thereby influencing the systemic toxicity and pharmacokinetics of drugs [2]. In eukaryotic cells, most efflux pumps, including P-glycoprotein (P-gp), transport substrates from the cytoplasm to the extracellular space or into intracellular compartments [3,4]. P-gp is a member of the ATP-Binding Cassette (ABC) transporter family and mediates the transport of numerous structurally unrelated compounds, including drugs, toxins, and endogenous metabolites. Given its important role in maintaining brain homeostasis and protecting the brain through efflux of toxic substances [4,5], alterations in P-gp expression and function have been proposed as a possible etiology of neurodegenerative diseases, including Alzheimer’s disease and Parkinson’s disease [4,6,7,8,9]. These alterations may be caused by stress, aging, inflammatory responses, genetic differences, and the administration of pharmaceuticals [4,10,11]. Indeed, it is hypothesized that altered P-gp transport at the level of the blood–brain barrier may be an important factor contributing to the accumulation of aggregated proteins in neurodegenerative diseases [10]. These observations have stimulated growing interest in the development of efflux pump modulators as potential therapeutic tools. In this context, Ibba et al. recently synthesized and chemically characterized a phenoxymethylquinoxaline derivative, 2,2′-(pyrido[2,3-g]quinoxaline-2,3-diylbis(methylene))bis(oxy)bis(N-phenylbenzamide), named **10d** [12]. This compound, used in this study, belongs to the phenoxymethylquinoxaline class, a family of efflux pump inhibitors previously shown to restore or potentiate the anticancer activity of chemotherapeutic agents in tumor cell lines [12,13,14,15] and to enhance the efficacy of antitubercular drugs against multidrug-resistant Mycobacterium tuberculosis strains [14] (Figure 1).

Although **10d** displays no intrinsic antitubercular activity, it significantly potentiates the activity of several antitubercular drugs [14]. Moreover, compared with established efflux pump inhibitors such as MK-571, Novobiocin and Verapamil, **10d** exhibited stronger and more selective inhibition of fluorescent dye efflux and the highest multidrug resistance (MDR) reversal activity when co-administered with the chemotherapeutic agents vincristine and etoposide at non-cytotoxic concentrations [12]. Considering the pivotal role of P-gp in maintaining neuronal homeostasis and regulating the brain exposure to endogenous and exogenous compounds, it is important to investigate the biological effects of novel efflux pump inhibitors in neuronal models. To date, however, the effects of **10d** on neuronal cells have not yet been explored. Therefore, in the present study we evaluated the effects of **10d** in PC12 cells, a well-established dopaminergic neuronal model widely used to investigate the molecular mechanisms underlying dopaminergic neurotoxicity [16,17]. We first assessed the direct effects of **10d** using an MTT-based cell viability assay. Since inhibition of efflux transporters could increase the intracellular accumulation of neurotoxic compounds, we then investigated whether **10d** modulates the toxicity induced by 1-methyl-4-phenyl-1,2,3,6-tetrahydropyridine (MPTP; 0.5–1 mM), a well-established neurotoxin widely employed to reproduce parkinsonian features in both in vitro and in vivo experimental models [18]. In addition, intracellular and extracellular dopamine (DA) levels, together with DA metabolites, were quantified by HPLC analysis to evaluate the impact of **10d** on dopaminergic homeostasis. Finally, to better understand the effect of **10d**, we evaluated its behavior in the presence of AMA, an antiparkinsonian drug used in clinical practice for the treatment of motor symptoms, known for its modulatory action on the dopaminergic system [19].

## 2. Materials and Methods

### 2.1. Cell Culture

PC12 cells derived from a pheochromocytoma cell line (ATCCCRL-1721, American Type Culture Collection (ATCC), Manassas, VA, USA) (passages 12–25) of the rat adrenal medulla were maintained at 37 °C in a 95% humidified atmosphere and 5% CO_2_ in 100 mm plastic culture dishes in Dulbecco’s Modified Eagle Medium (DMEM)/F12 (Catalog number: 11039021, Gibco™, Thermo Fisher SCIENTIFIC: Life Technologies Italia Fil., Monza, MB, Italy) supplemented with 10% horse serum, 5% fetal bovine serum, and 1% penicillin/streptomycin, as reported by Migheli et al. [20].

### 2.2. Drug Treatments

PC12 cells were treated for 24, 48 and 72 h with **10d** (0.1, 0.5, 1, 5 and 10 µM) alone (control) or with MPTP (M0896, Sigma-Aldrich, Merck Life Science S.r.l., Milan, Italy) (0.5 and 1 mM), administered 20 min after **10d**) **10d** (10 µM), alone or with MPTP (0.75 mM) at 24 h and AMA (A1260, Sigma-Aldrich, Merck Life Science S.r.l., Milan, Italy) (500µM). The solutions of both substances were prepared in DMEM/F12 medium. All experiments were performed in triplicate. The MPTP concentration (1 mM) at 24 h was chosen based on the literature [18]. Lower concentrations than 1 mM of MPTP have been used for a longer time [21]. The concentrations of **10d** used in the present study were selected based on the previous work by Ibba et al. [12], in which the compound was evaluated over a concentration range of 0.1–10 μM for its biological activity as a P-gp inhibitor. To ensure consistency with the published pharmacological profile of the compound, we initially tested the same concentration range in our MTT assays. Based on these preliminary experiments, we selected the 5 and 10 μM concentrations, as they produced significant effects in PC12 cells and therefore represented the most appropriate concentrations for investigating the interaction between **10d** and MPTP-induced neurotoxicity.

### 2.3. Cell Viability Measurement by MTT Assay

Cell viability was assessed with the 3-(4, 5-dimethyl-thiazol-2- yl)−2, 5, diphenyltetrazolium bromide (MTT; Sigma-Aldrich, Merck Life Science S.r.l., Milan, Italy) assay in which only viable cells convert the soluble dye MTT in soluble (in aqueous media) blue formazan crystals by the action of mitochondrial reductase.

In brief, at the end of the exposure time, 100 uL of MTT (0.65 mg/mL) was added to each sample and incubated for 2 h at 37 °C. After the incubation the MTT solution was removed, and 100 uL of DMSO (dymethil-sulfoxide, 1.02950.0500, Supelco, Merck Life Science S.r.l., Milan, Italy) was added and the plate was gently shaken before the detection of the absorbance. The absorbance value was detected by a Beauty Diagnostic Microplate Reader (SpectraMax 190, Molecular Devices, Wokingham, Berkshire, UK) at 578 nm. All experiments were performed in 96-well plates (5 × 10^3^ cells/well) and repeated in triplicate, as reported by Rassu et al. [22,23]. All experiments were performed in triplicate.

### 2.4. Chromatographic Analysis

Samples for HPLC analysis were prepared as follows: PC12 content of a confluent Petri dish (ca. 5 × 106 cells) was collected, washed three times with phosphate-buffered saline (PBS), and centrifuged at 700 rpm for 5 min at r.t. After removal of the supernatant, the cell pellet was resuspended in 350 μL of ice-cold meta-phosphoric acid (m-H_3_PO_4_ 0.1 M) and sonicated to ensure complete cell lysis and the extraction of intracellular metabolites. A 20 μL aliquot of the resulting homogenate was used for protein determination using the Bradford assay. The remaining homogenate was centrifuged at 10,000× *g* for 10 min at r.t., and the resulting supernatant was collected for chromatographic analysis. A 30 μL aliquot was injected into the HPLC system for electrochemical detection. L-DOPA, DA, 3,4-dihydroxyphenylacetic acid (DOPAC), homovanillic acid (HVA), and 3-methoxytyramine (3-MT) were quantified in experiments by High-Pressure Liquid Chromatography Electrochemical detection (HPLC–EC), using an Alltech 426 HPLC pump (Alltech, Sedriano, Italy) equipped with a Rheodyne injector (model 7725, Rohnert Park, CA, USA), a column (15 cm × 4.6 mm i.d., ODS80TM C18, Toso Haas, Stuttgart, Germany), an electrochemical detector ANTEC–Leyden EC controller (ANTEC, Zoeterwoude, The Netherlands), and a PC-based ADC system (Varian Star Chromatographic Workstation, Varian, Walnut Creek, CA, USA). The mobile phase was citric acid (0.1 M), ethylenediaminetetraacetic acid (EDTA, 1.0 mM), MeOH (8.7%), and sodium octylsulfate (48 mg/L), with a flow rate of 1.2 mL/min and pH 2.9 [24,25].

### 2.5. Statistical Analysis

All experimental data are expressed as mean values ± SEM. In the presence of overall significant main effects and interactions (*p* values < 0.05) following one-way analysis of variance (ANOVA), a Bonferroni multiple comparisons test was performed as a post hoc analysis. All data were evaluated by using Graph-Pad Prism 10.0 software (Inc., San Diego, CA, USA).

## 3. Results

Statistical analyses were performed using one-way or two-way ANOVA, followed by Tukey’s or Bonferroni’s multiple comparison tests, respectively. All statistical details, including F-values and *p*-values, are reported in the corresponding figure legends.

### 3.1. Effects of **10d** and MPTP (0.5–1 mM) at Different Time Points on MTT-Based Cell Viability Assay

**Figure 2:**
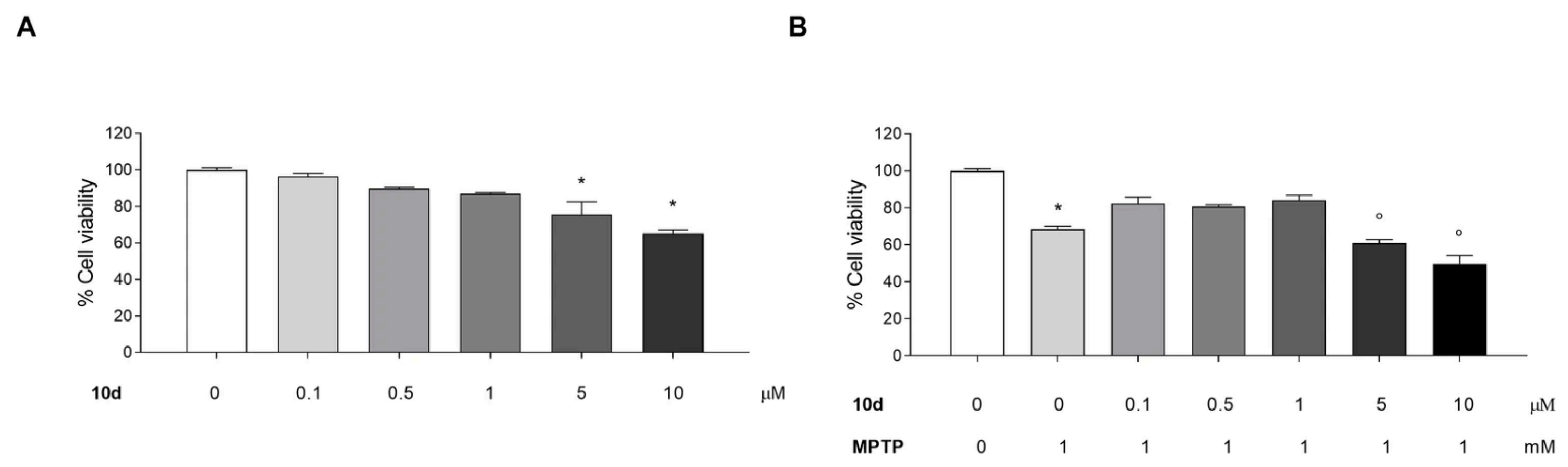
Screening of different concentrations of compound **10d** was performed at 24 h (panel (**A**)), and the corresponding co-treatment with 1 mM MPTP is shown in panel (**B**). Data are expressed as mean ± SD. Statistical analysis was performed using one-way ANOVA, followed by Tukey’s multiple comparisons test. (* *p* < 0.05 vs. CTRL; ° *p* < 0.05 vs. MPTP). Panel (**A**): F(5,12) = 54.75, *p* < 0.0001; panel (**B**): F(6,14) = 124.5, *p* < 0.0001.

**Figure 3:**
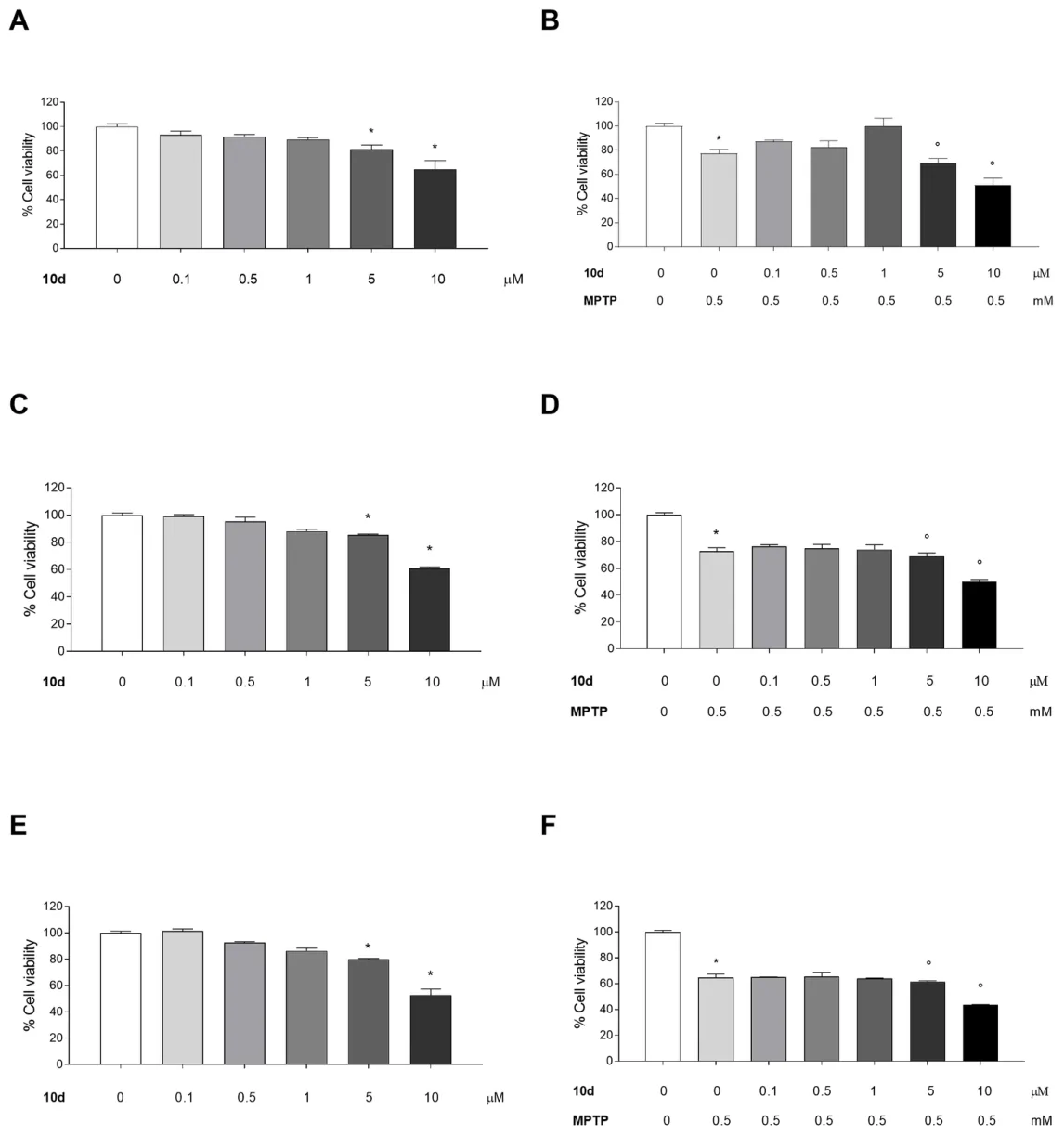
**10d** was evaluated at 24 h (panel (**A**)), 48 h (panel (**C**)), and 72 h (panel (**E**)), while the corresponding co-treatment with 0.5 mM MPTP is shown in panels (**B**) (24 h), (**D**) (48 h), and (**F**) (72 h). Data are expressed as mean ± SD. Statistical analysis was performed using one-way ANOVA, followed by Tukey’s multiple comparisons test. (* *p* < 0.05 vs. CTRL; ° *p* < 0.05 vs. MPTP). Panel (**A**): F(5,12) = 32.53, *p* < 0.0001; panel (**B**): F(6,14) = 45.14, *p* < 0.0001; panel (**C**): F(5,18) = 312.1, *p* < 0.0001; panel (**D**): F(6,21) = 150.1, *p* < 0.0001; panel (**E**): F(5,12) = 189.0, *p* < 0.0001; panel (**F**): F(6,14) = 280.9, *p* < 0.0001.

Compound **10d**, tested alone for 24, 48, and 72 h, does not elicit any significant toxicity in PC12 cells at the lowest concentrations. Indeed, cytotoxicity is evident only at the highest concentrations tested (5 and 10 μM), as can be seen from Figure 2A and Figure 3A,C,E.

To further investigate the properties of this extrusion pump inhibitor, we decided to test it in a cell culture model of experimental parkinsonism, induced by MPTP at the concentrations of 1 mM and 0.5 mM at different time points (Figure 2B and Figure 3B,D,F) [18]. As can be observed from Figure 2B and Figure 3B,D,F, compound **10d** worsens the cytotoxicity of MPTP at the same two highest concentrations. In particular, after 24 h of exposure to 1 mM MPTP, cell viability undergoes a significant decrease (68%) compared with the control, and concomitant treatment with **10d** at the two highest concentrations tested further reduces it (to 61% and 50%, respectively) with respect to the MPTP group. Under the same experimental conditions, 0.5 mM MPTP induces a higher reduction in cell viability after 24, 48 and 72 h if co-administered with **10d** at the highest concentrations. In particular, the cell viability rates after MPTP treatment at 24, 48 and 72 h are 77%, 72% and 65%, respectively, whereas the values after MPTP + **10d** treatment at 5 μM are 69%, 69% and 62%, and after MPTP + **10d** treatment at 10 μM are 51%, 50% and 44%.

### 3.2. Effects of **10d** and MPTP (1 mM) at 24 h on Intracellular DA and DA Metabolite Levels

To better investigate the effect of **10d**, we evaluated any changes in DA and DA metabolite levels following its administration alone and in combination with 1 mM MPTP at 24 h. The experiments were conducted using **10d** at the highest concentrations selected from the screening.

**Figure 4:**
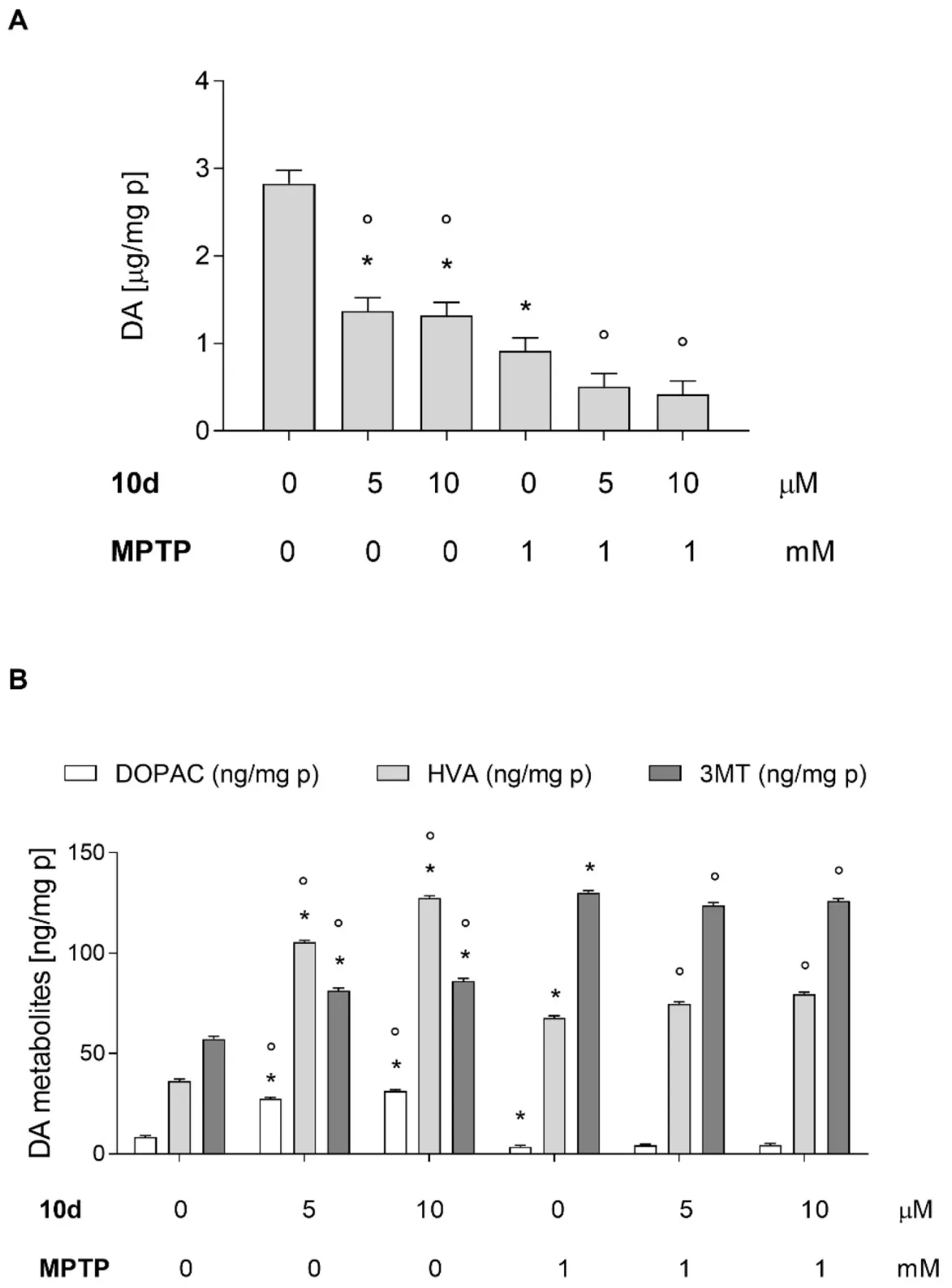
Intracellular DA (panel (**A**)) and DA metabolite (panel (**B**)) levels in PC12 cells after treatment with **10d** and 1 mM MPTP at 24 h; * *p* < 0.05 vs. control; ° *p* < 0.05 vs. MPTP. Data are expressed as mean ± SD. Statistical analysis was performed using one-way ANOVA, followed by Tukey’s multiple comparisons test for intracellular DA levels, and using two-way ANOVA for DA metabolite levels, followed by Bonferroni’s multiple comparisons test. Panel (**A**): F(5,12) = 102.3, *p* < 0.0001; panel (**B**): two-way ANOVA revealed a significant main effect of treatment (F(2,36) = 12,713.96, *p* < 0.0001).

Figure 4A shows that the intracellular DA levels are significantly reduced by 5 (1.371 μg/mg p) and 10 μM (1.318 μg/mg p) **10d** after 24 h of exposure in PC12 cells. MPTP 1 mM significantly reduces levels of DA (0.916 μg/mg p) with respect to the control and to a greater extent than the 5 and 10 μM concentrations **10d**. In addition, the MPTP 1 mM- induced reduction in DA levels is significantly higher if MPTP was co-administered with **10d** at the concentrations 5 (0.502 μg/mg p) and 10 μM (0.420 μg/mg p) with respect to MPTP alone. Figure 4B shows the intracellular metabolites of DA (DOPAC, HVA, 3MT) release measured after 24 h from **10d** incubation (5 and 10 μM). In this place both concentrations of **10d** increased DOPAC (27.5 and 31.3 ng/mg p), HVA (105.6 and 127.6 ng/mg p) and 3MT (81.4 and 86.13 ng/mg p) levels with respect to the corresponding basal concentration in the control group (8.4; 36.3 and 57.4 ng/mg p). As shown in panel B, MPTP increases DA metabolite (HVA, 3MT) levels (67.85 and 130 ng/mg p) except for DOPAC (3.5 ng/mg p), with respect to the corresponding basal concentration in the control group. Both concentrations of **10d** (5 and 10 μM) in association with MPTP alter DA metabolites. In particular, **10d** increases HVA levels with respect to the corresponding metabolites concentration in the MPTP group. By contrast the same concentrations of **10d** reduces 3MT levels with respect to the MPTP group.

### 3.3. Effects of **10d** and MPTP (0.5 mM) at 48 h on Intracellular DA and DA Metabolite Levels

**Figure 5:**
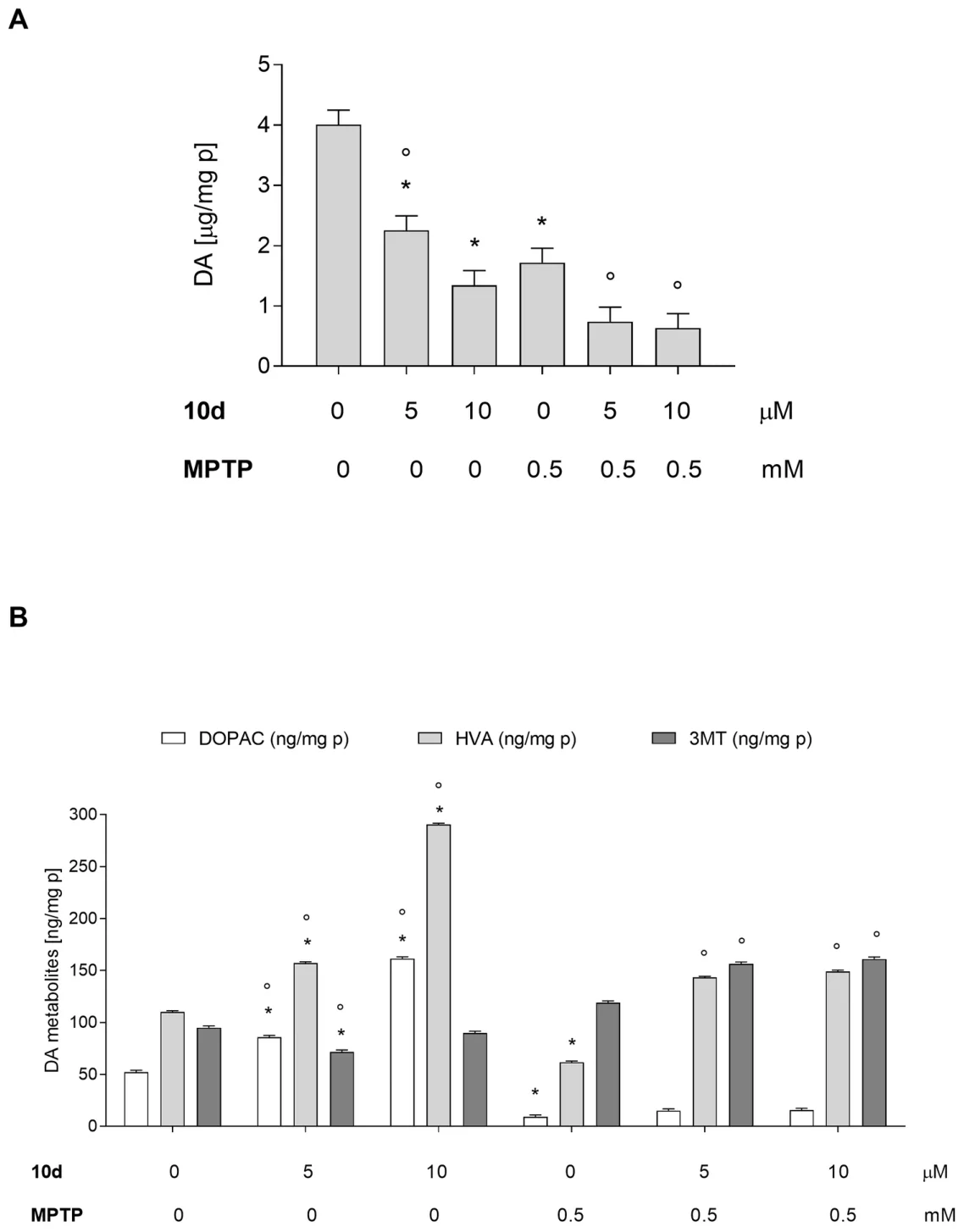
Intracellular DA (panel (**A**)) and DA metabolite (panel (**B**)) levels in PC12 cells after treatment with **10d** and 0.5 mM MPTP for 48 h. * *p* < 0.05 vs. control; ° *p* < 0.05 vs. MPTP. Data are expressed as mean ± SD. Statistical analysis was performed using one-way ANOVA, followed by Tukey’s multiple comparisons test for intracellular DA levels, and using two-way ANOVA for DA metabolite levels, followed by Bonferroni’s multiple comparisons test. Panel (**A**): F(7,8) = 277.9, *p* < 0.0001; panel (**B**): two-way ANOVA revealed a significant main effect of treatment (F(7,80) = 3963.74, *p* < 0.0001).

As reported in Figure 5A, **10d** at both concentrations significantly reduced intracellular DA levels compared to control (2.25 and 1.34 μg/mg p compared to 4.00 μg/mg p). MPTP 0.5 mM significantly reduced levels of DA (1.72 μg/mg p) with respect to the control and to **10d** 5 μM, but not to **10d** 10 μM. In addition, the reduction in DA levels induced by MPTP 0.5 mM was significantly higher if MPTP is associated with **10d** at 5 (0.74 μg/mg p) and 10 μM (0.63 μg/mg p) with respect to MPTP alone.

The intracellular DOPAC and HVA levels (Figure 5B) are significantly increased after treatment with **10d** 5 μM (DOPAC: 85.8 ng/mg p; HVA: 157.2 ng/mg p) and 10 μM (DOPAC: 161.4 ng/mg p; HVA: 290.5 ng/mg p) compared to the control (DOPAC: 52.2 ng/mg p; HVA: 110.4 ng/mg p). On the contrary, **10d** at the lowest concentration (5 μM) significantly reduced 3MT levels (71.7 ng/mg p), but it does not induce significant changes at the highest concentration (10 μM; 89.9 ng/mg p) with respect to the control (95.00 ng/mg p). MPTP 0.5 mM at 48 h significantly reduced the intracellular level of the metabolites DOPAC (9.3 ng/mg p) and HVA (61.7 ng/mg p) with respect to the control. 3MT levels increased after treatment with MPTP (119 ng/mg p) if compared to **10d** 5 μM, but not to **10d** 10 μM. In addition, the levels of HVA, 3MT were higher if MPTP was associated with different concentrations of **10d** with respect to MPTP alone. DOPAC levels did not have any changes (Figure 5B).

### 3.4. Effects of **10d** (10 μM), AMA (500 μM), and MPTP (0.75 mM) at 24 h on Intracellular DA and DA Metabolite Levels

The graph (panel A) shows that AMA alone significantly reduces intracellular DA levels (1.6 μg/mg p) compared to the control (2.045 μg/mg p), but not as much as **10d** (1.4 μg/mg p), which confirms its toxic effect on PC12 cells at the highest concentration. MPTP alone, used at the middle concentration 0.75 mM, induces a strong significant reduction in DA levels (0.71 μg/mg p) with respect to control, **10d** and AMA. AMA + MPTP + **10d** show a further decrease in DA levels (0.31 μg/mg p) with respect to control, MPTP, AMA + **10d** and AMA + MPTP.

Intracellular DOPAC (panel B) increases following treatment with **10d**, AMA, and AMA + **10d** (53.5 ng/mg p, 35.7 ng/mg p, and 53.4 ng/mg p, respectively), compared to the control (28.3 ng/mg p) and MPTP. It instead decreases in MPTP (11.36 ng/mg p) and AMA + MPTP (9.9 ng/mg p) groups with respect to the control. Intracellular HVA levels significantly increase in each experimental condition if compared to the control and to MPTP. HVA increases after treatment with AMA + MPTP + **10d** (193.8 ng/mg p) also with respect to AMA + MPTP (136.2 ng/mg p). 3MT increases in all experimental groups except to AMA alone (20.5 ng/mg p) with respect to the control (23.6 ng/mg p). It also increases after treatment with **10d** (29.8 ng/mg p), AMA + **10d** (36.1 ng/mg p), AMA + MPTP (46.8 ng/mg p) and AMA + MPTP + **10d** (69.5 ng/mg p) with respect to MPTP. Additionally, its intracellular levels are significantly reduced in the case of treatment with AMA with respect to MPTP.

## 4. Discussion

The present study is the first to investigate the biological effects of the pyridoquinoxaline-based P-gp inhibitor **10d** in a dopaminergic neuronal model. Our findings demonstrate that **10d** reduces PC12 cell viability and significantly potentiates MPTP-induced cytotoxicity, suggesting that inhibition of efflux pump activity increases neuronal susceptibility to toxic insults. Moreover, **10d** markedly altered dopamine (DA) homeostasis by reducing intracellular DA levels and modifying the profile of DA metabolites. Finally, the combined exposure to **10d**, MPTP and amantadine produced a greater disruption of dopaminergic metabolism than each compound alone, highlighting the potential role of efflux pump activity in regulating neuronal vulnerability and the cellular response to antiparkinsonian drugs.

Our findings are consistent with the growing evidence supporting a neuroprotective role of efflux pumps and, in particular, P-glycoprotein (P-gp). Besides its well-established involvement in multidrug resistance, P-gp represents a key component of the cellular detoxification system, limiting the intracellular accumulation of xenobiotics and endogenous toxic compounds. Bartels et al. proposed the “neurotoxin hypothesis”, suggesting that impaired detoxification mechanisms may increase susceptibility to Parkinson’s disease (PD). In this context, both P-gp and multidrug resistance-associated proteins (MRPs) have been recognized as essential components of the brain defense system, preventing the penetration and accumulation of potentially neurotoxic molecules [7,26]. Therefore, pharmacological inhibition of MRPs may compromise these protective mechanisms, increasing neuronal vulnerability.

Consistent with this hypothesis, treatment with **10d** alone moderately reduced PC12 cell viability, while its combination with MPTP produced a significantly greater cytotoxic effect. One possible explanation is that inhibition of P-gp-mediated transport may reduce the cellular capacity to eliminate neurotoxic compounds, thereby enhancing their intracellular retention and toxic effects. This interpretation agrees with the established protective function of P-gp against xenobiotics and supports the hypothesis that alterations in efflux transporter activity may contribute to neuronal degeneration.

In addition to affecting cell viability, **10d** influenced dopaminergic homeostasis. Treatment with the efflux pump inhibitor significantly reduced intracellular DA levels, an effect that became even more pronounced following co-treatment with MPTP. A plausible explanation is that inhibition of cellular efflux mechanisms alters DA compartmentalization, increasing the cytosolic pool of dopamine available for oxidative metabolism [27]. Elevated cytosolic DA is particularly susceptible to monoamine oxidase (MAO)-mediated deamination, generating DOPAC together with hydrogen peroxide, thereby promoting oxidative stress [28]. Mosharov et al. demonstrated that increased cytosolic DA is intrinsically toxic because it undergoes rapid oxidation and degradation, ultimately contributing to dopaminergic neuronal damage [29]. Therefore, the reduction in intracellular DA observed in our study likely reflects accelerated DA turnover associated with increased oxidative metabolism rather than simply a reduction in DA synthesis.

The analysis of DA metabolites further supports this interpretation. After 24 h of treatment, **10d** alone induced an increase in all three DA metabolites, suggesting enhanced dopamine catabolism. Conversely, the combination of **10d** with MPTP resulted in decreased DOPAC and HVA levels, together with increased 3-methoxytyramine (3-MT), indicating a shift in dopamine metabolism that may reflect preferential catechol-O-methyltransferase (COMT) activity over MAO. Similar biochemical alterations have previously been reported in experimental models of PD. Kuroiwa et al. described significant reductions in striatal DA, DOPAC and HVA following MPTP administration in mice [30], whereas Strekalova et al. reported increased 3-MT levels associated with impaired vesicular DA storage and progressive degeneration of dopaminergic terminals [31]. Our findings are therefore consistent with previous reports indicating that MPTP disrupts dopamine metabolism while altering the balance between its major metabolic pathways.

Interestingly, after 48 h, although intracellular DA remained significantly reduced following co-treatment with **10d** and MPTP, the increase in 3-MT observed at 24 h was no longer evident. This temporal difference suggests the activation of compensatory metabolic mechanisms following the initial toxic insult. In agreement with this hypothesis, Kolacheva et al. demonstrated that MPTP induces time-dependent adaptations in dopamine metabolism accompanied by progressive reductions in tyrosine hydroxylase activity and DA content [32,33]. Taken together, these findings suggest that the lack of a sustained increase in 3-MT at 48 h reflects partial metabolic re-equilibration resulting from reduced substrate availability, together with adaptive changes in MAO- and COMT-dependent pathways.

**Figure 6:**
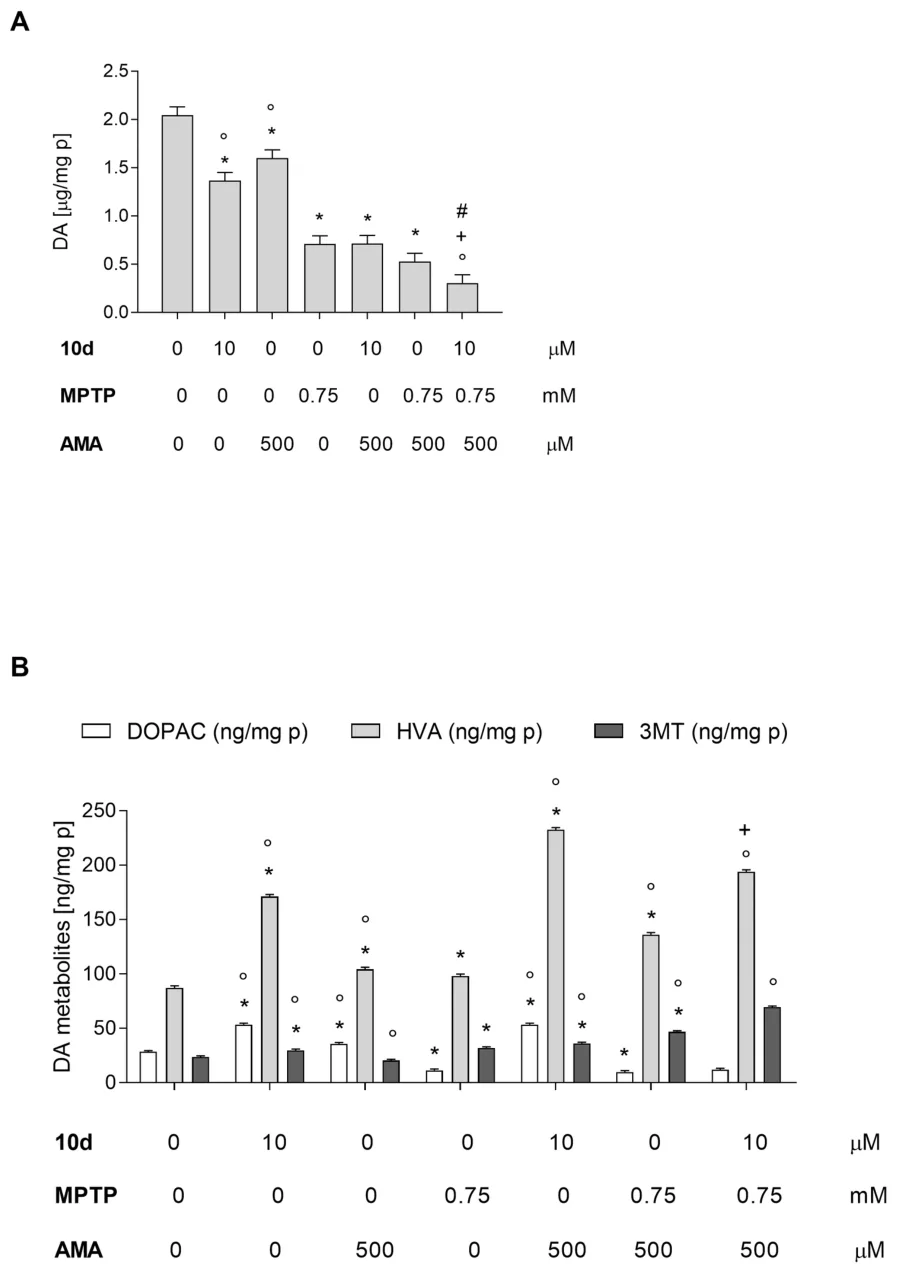
Intracellular DA (panel (**A**)) and DA metabolite (panel (**B**)) levels in PC12 cells after treatment with **10d**, AMA, and 0.75 mM MPTP at 24 h. * *p* < 0.05 vs. control; ° *p* < 0.05 vs. MPTP; + *p* < 0.05 vs. AMA + MPTP; # *p* < 0.05 vs. AMA + **10d**. Data are expressed as mean ± SD. Statistical analysis was performed using one-way ANOVA, followed by Tukey’s multiple comparisons test for intracellular DA levels, and using two-way ANOVA for DA metabolite levels, followed by Bonferroni’s multiple comparisons test. Panel (**A**): F(9,10) = 1008, *p* < 0.0001; panel (**B**): two-way ANOVA revealed a significant main effect of treatment (F(4,100) = 51,383.71, *p* < 0.0001).

Another important finding of the present study is the interaction between **10d** and amantadine (AMA). Although AMA is used as an antiparkinsonian drug, our results indicate that its combination with **10d** and MPTP produced a more pronounced reduction in intracellular DA than each treatment alone. Rather than acting through a single pathway, these compounds appear to converge on complementary mechanisms that synergistically impair dopaminergic homeostasis. AMA increases extracellular DA by promoting dopamine release and limiting its reuptake. The resulting decrease in intracellular dopamine levels (Figure 6A) was accompanied by significant alterations in dopamine metabolite levels (Figure 6B), indicating a marked perturbation of intracellular dopaminergic homeostasis. In parallel, MPTP is converted into MPP^+^, which selectively inhibits mitochondrial complex I, causing mitochondrial dysfunction, ATP depletion and increased reactive oxygen species generation [34]. Inhibition of P-gp by **10d** may further exacerbate these events by reducing the cellular capacity to eliminate toxic metabolites and oxidative by-products. The convergence of these mechanisms may amplify oxidative stress, impair vesicular dopamine storage and ultimately accelerate dopaminergic neuronal dysfunction [35,36,37]. Although the present findings provide novel insights about the effects of **10d** on dopaminergic cells, this study should be considered preliminary. Further in vivo studies will be required to validate these observations and to assess their potential translational relevance.

## 5. Conclusions

Collectively, our findings identify that **10d** could be considered an amplifier of dopaminergic vulnerability. At the highest concentrations, the compound exacerbates MPTP-induced cytotoxicity and drives a significant depletion of intracellular DA by shifting its distribution toward the oxidative cytosolic pool. The dynamic remodeling of DA metabolism further reflects an early but insufficient adaptive response to mitochondrial dysfunction. Remarkably, the combined exposure to AMA, MPTP, and **10d** prompts a synergistic collapse of dopaminergic homeostasis, showing a self-amplifying cycle of oxidative stress and impaired detoxification. Although these findings are limited to an *in vitro* model, and P-gp expression may vary among PC12 cell clones, they raise the possibility that inhibition of efflux transporters may influence the cellular response to dopaminergic neurotoxins. However, the molecular mechanisms underlying these effects remain to be elucidated. Future studies should evaluate the expression of P-gp and other relevant efflux transporters and conduct *in vivo* studies to further assess the physiological relevance of the present findings.

## Data Availability

The original contributions presented in this study are included in the article. Further inquiries can be directed to the corresponding author.

## Abbreviations

The following abbreviations are used in this manuscript:

P-gp	P-glycoprotein
**10d**	2,2′-((pyrido[2,3-g]quinoxaline-2,3-diylbis(methylene))bis(oxy))bis(N-phenylbenzamide)
DA	dopamine
MPTP	N-methyl-4-phenyl- 1,2,3,6-tetrahydropyridine
AMA	Amantadine
DOPAC	3,4-dihydroxyphenylacetic acid
HVA	Homovanillic acid
3-MT	3-methoxytyramine
HPLC	High-Pressure Liquid Chromatography
